# Beyond translations, perspectives for researchers to consider to enhance comprehension during consent processes for health research in sub-saharan Africa: a scoping review

**DOI:** 10.1186/s12910-023-00920-1

**Published:** 2023-06-21

**Authors:** Nkosi Busisiwe, Janet Seeley, Ann Strode, Michael Parker

**Affiliations:** 1 Africa Health Research Institute, KwaZulu-Natal Durban, South Africa; 2 Institute for Global Health, University College London, London, UK; 3 School of Law, University of KwaZulu-Natal, Pietermaritzburg, South Africa; 4 London School of Hygiene and Tropical Medicine, London, UK; 5 School of Nursing and Public Health, University of KwaZulu-Natal, Durban, South Africa; 6 South African Research Ethics Training Initiative, University of KwaZulu-Natal, Pietermaritzburg, South Africa; 7 Ethox Centre, University of Oxford, Oxford, UK

**Keywords:** Comprehension, Informed consent, Language, Sub-saharan Africa, Translations

## Abstract

**Background:**

Literature on issues relating to comprehension during the process of obtaining informed consent (IC) has largely focused on the challenges potential participants can face in understanding the IC documents, and the strategies used to enhance comprehension of those documents. In this review, we set out to describe the factors that have an impact on comprehension and the strategies used to enhance the IC process in sub-Saharan African countries.

**Methods:**

From November 2021 to January 2022, we conducted a literature search using a PRISMA tool. We searched electronic databases (PubMed, EMBASE, EBSCOHOST) to identify relevant peer reviewed studies. We then reviewed the references of these articles to find additional literature that might have been missed through the initial search. We were particularly interested in full text articles in English that focused on the IC process in SSA published between 2006 and 2020. We included systematic reviews, and studies from Western and Asian countries that included data about SSA. We excluded articles that focused on medical interventions and studies that did not require IC.

**Results:**

Out of the 50 studies included most were multi-country (*n* = 13) followed by single country studies in South Africa (*n* = 12); Kenya, Tanzania, Uganda (*n* = 5) each; Gambia, Ghana and Nigeria (*n* = 2)each ; and one each for Botswana, Malawi, Mali, Mozambique. We identified three areas of focus: (1) socio-cultural factors affecting IC; (2) gaps in the ethical and legal frameworks guiding the IC process; and (3) strategies used to improve participants’ understanding of IC.

**Conclusion:**

Our review showed wide recognition that the process of achieving IC in SSA is inherently challenging, and there are limitations in the strategies aimed at improving comprehension in IC. We suggest that there is a need for greater flexibility and negotiation with communities to ensure that the approach to IC is suited to the diverse socio-cultural contexts. We propose moving beyond the literal translations and technical language to understanding IC comprehension from the participants’ perspectives and the researchers’ views, while examining contextual factors that impact the IC process.

**Supplementary Information:**

The online version contains supplementary material available at 10.1186/s12910-023-00920-1.

## Background

The achievement of valid IC is universally recognized as central to the ethical conduct of scientific research [1–3]. However, ensuring valid consent is complex for a number of reasons, including, balancing the differing interests of the participants, researchers and sponsors in a single document [1–3], facilitating informed choices by participants [1–3], meeting ethical and legal obligations and applying them in local contexts [4] and addressing the impact of real world settings.

These issues can be clustered around two key problems, disjuncture between the national and international legal-ethical stipulations [4], and the limited guidance on obtaining informed consent in social and cultural contexts where decision-making is not solely in the hands of an individua [1, 4–6]. The use of poorly designed IC documents, misunderstanding in local languages and terms used in IC documents, and low literacy levels result in poor understanding during the IC process [7, 8]. Although poorly designed IC materials is a global problem, low-and middle income countries, (LMIC) including sub-Saharan (SSA) countries face unique challenges. These range from how to address beliefs about health and decision making to views about autonomy, and low functional literacy levels in English. The literature increasingly shows that alternative approaches are needed to address this issue in LMIC. Some work has been done in this regard with attention being placed on the translation of IC documents into local languages, the development of tools designed to enhance participants’ understanding [1, 4], and tailoring the IC information to suit local contexts [5].

Against this background we set out to explore topics focusing on comprehension of the IC process across SSA countries. We also describe and highlight the challenges of strategies used to enhance the process of understanding IC in SSA. Although there are many ways in which consent processes can fail to achieve valid consent, our focus in this review was on comprehension. We propose moving beyond the literal translations and technical language to understanding IC comprehension from the participants’ perspectives and the researchers’ views, while examining contextual factors that impact the IC process.

### Objectives

1. Identify studies focusing on exploring obstacles to comprehension and relevant strategies to enhance the understanding of IC documents in SSA, paying particular attention to language and translations.
2. Describe strategies to enhance comprehension during the informed consent process in SSA countries with diverse local settings.
3. Suggest perspectives for researchers to consider to enhance comprehension of the informed consent process in diverse settings in SSA countries.

## Methods

### Study selection

From November 2021 to January 2022, we conducted a literature search using a PRISMA tool.” We were interested in articles that focused on the IC process in SSA. In the first stage, we searched the electronic databases (PubMed, EMBASE, EBSCOHOST) to identify peer reviewed studies. We then manually reviewed the references of these articles for additional relevant literature that might have been missed through the initial search. We conducted searches using a combination of the following terms: ‘informed consent’ or ‘comprehension’ or’ health research’, or “language” or translation’ or ‘sub-Saharan Africa’. Furthermore, we searched various individual SSA countries to ensure that we included articles which we may have missed when using the term “sub-Saharan Africa” (Table 1: Search strategy and selection criteria).

**Table 1:** Search strategy and selection criteria

Level	Components of search
Level 1	Search engines: PubMed, EMBASE, EBSCOHOST, Google Scholar Key words: ‘informed consent’ or ‘comprehension’ or ‘health research’, or “language” or translation’ or ‘sub-Saharan Africa’ Key words + name of the country
Level 2	Manual search of studies identified in Level 1

### Inclusion criteria

We included articles focusing on the process of informed consent in (1) multidisciplinary fields including social sciences, medical research and bio banking; (2) studies focusing on broader environmental issues that impact the IC process such as the socio-cultural factors and the ethical and legal frameworks that govern the IC process; and (3) studies focusing on strategies aimed at improving the comprehension of IC process including issues around translations, language and its meaning, and IC comprehension assessments. We also included systematic reviews, and studies from Western, and Asian countries that included data about SSA countries. We included full text English articles published between 2006 and 2020. This period was critical because of growth in clinical research especially HIV and AIDS, as well as increase in global health research which highlighted differences and anomalies during the informed consent process between global south and global north.

### Exclusion criteria

We excluded articles that focused on medical interventions and studies that did not require IC. We also excluded studies that were not conducted in SSA.

### Study selection

We use the Preferred Reporting Items for Systematic Reviews and Meta-Analyses (PRISMA) tool to guide the selection process. The initial search resulted in a total of 66,310 articles from the electronic search, and (*n* = 9) from directed search. Records initially identified through the search were screened to exclude studies that were unrelated to the research topic. After these irrelevant studies (*n* = 66,122) were excluded, the titles and abstracts of 189 articles were screened by authors BN, JS and MP to determine applicability according to inclusion and exclusion criteria. A total of 72 articles were rejected after the titles and abstracts were reviewed. The studies were screened independently, and later as a team.

**Fig. 1:**
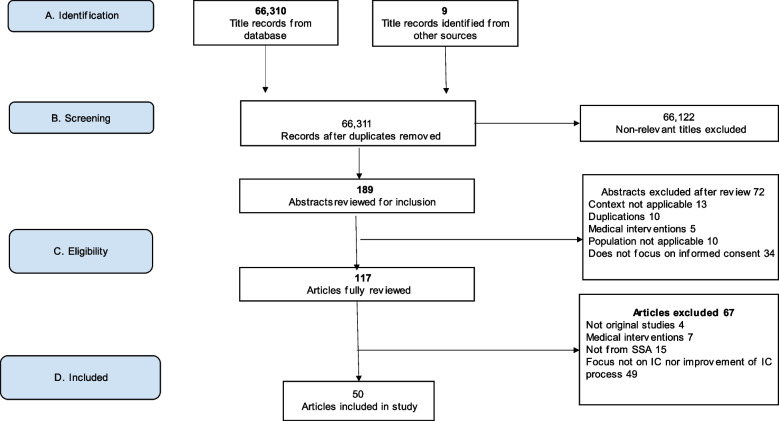
Flow chart of the search process

Differences and discrepancies were resolved by discussion until consensus was reached. Following this level of screening, full text of 117 articles were screened, and 67 articles were excluded using the same process. A total of 50 articles met the criteria and were included for review, Fig. 1: Flow chart of the search process. Of the 50 studies, 44 were primary studies and 6, systematic review studies.

We used the framework thematic analysis approach to develop and organize the themes [9]. Using a matrix (Word document) BN, JS and MP categorised articles that met the criteria into 3 main focus areas, (1) gaps in the ethical and legal frameworks guiding informed consent process); (2) socio-cultural factors; and (3) strategies used to improve participants’ understanding of the IC. (Supplementary Table 1: Thematic analysis of the studies reviewed). Studies that addressed more than one theme were clustered into one category using a consensus approach.

Out of the 50 studies included in the review (Table 2, Summary of reviewed studies) most were multi-country studies (*n* = 13) followed by single country studies in South Africa (*n* = 12); Kenya, Tanzania, Uganda (*n* = 5) respectively; Gambia, Ghana and Nigeria (*n* = 2) respectively; and Botswana, Malawi, Mali, and Mozambique one each. All studies reviewed show that the IC process is fraught with challenges and complexities across SSA. Most of the studies focused on adults, with two focusing on IC in paediatric research [10, 11]. We identified three areas which impact on the IC process, namely, (1) socio-cultural factors; gaps in the ethical and legal frameworks guiding the IC process); (2) and (3) strategies used to improve participants’ understanding of the IC.

**Table 2:** Summary of reviewed studies

Author	Publication Date	Title	Country	Focus area	Journal
Afolabi, M.O., Bojang, K. D’Alessandro, U. et al.	2014	Digitised audio questionnaire for” assessment of informed consent comprehension in a low-literacy African research population: Development and psychometric evaluation	Gambia	Assessment tools for” IC comprehension Translation of IC documents	BMJ Open
Afolabi, Muhammed O. Okebe, Joseph U. Mcgrath, Nuala Larson, Heidi J. Bojang, Kalifa Chandramohan, Daniel	2014	Informed consent comprehension in African research settings	Multi-country	Measurement of IC comprehension	Tropical Medicine and International Health
Afolabi, Muhammed Olanrewaju Rennie, Stuart Halfords, Denise Dion Kline, Tracy Zeitz, Susannah Odongo, Frederick S. Amek, Nyaguara O. Luseno, Winnie K.	2018	An adapted instrument to assess informed consent comprehension among youth and parents in rural western Kenya: a validation study	Kenya	Development of a cross-cultural tool for” the adaptability and validation of an informed consent	BMJ open
Akpa-Inyang, Francis Chima, Sylvester C.	2021	South African traditional values and beliefs regarding informed consent and limitations of the principle of respect for” autonomy in African communities: a cross-cultural qualitative study	South Africa	Perceptions of biomedical researchers regarding the comprehensibility of the informed consent doctrine and its application in African countries.	BMC Medical Ethics
Marshall, P.A	2006	Informed Consent in International Health Research	Multi-country	Socio-cultural factors that influence comprehension of IC. Recommendations for good practice	A Journal of Empirical Research on Human Research Ethics: An International Journal
Appiah, Richard	2021	Gurus and Griots: Revisiting the research informed consent process in rural African contexts	Ghana	Strategies for” context-appropriate ethical guidelines to improve IC process in African rural settings	BMC Medical Ethics
Barchi, Francis Little, Madison T.	2016	National ethics guidance in SSA on the collection and use of human biological specimens: A systematic review Ethics in Biomedical Research	Multi-country	Availability of national ethics and regulatory guidance on biomedical research in Sub- Saharan African countries and	BMC Medical Ethics
Bentley, Margaret E Sorenson, James R Henderson, Gail E Horst, Charles Van Der Moses, Agnes Nkhoma, Jacqueline Ahmed, Yusuf	2011	Using formative research to develop a context-specific approach to informed consent for clinical trials	Malawi	Development of context-specific consent information	
Boga, Mwanamvua Davies, Alun Kamuya, Dorcas Kinyanjui, Samson M. Kivaya, Ester Kombe, Francis, et al.	2011	Strengthening the informed consent process in international health research through community engagement: The KEMRI-Wellcome Trust Research Programme experience	Kenya	Strategies for” strengthening the informed consent – developing dictionary of terminologies and community engagement.	PLoS Medicine
Britz, Retha le Roux-Kemp, Andra	2016	Voluntary informed consent and good clinical practice for” clinical research in South Africa: Ethical and legal perspectives	South Africa	Review of the legal and ethical guidelines on informed consent	S Afr Med Journal
Bukini, Daima Mbekenga, Columba Nkya, Siana Purvis, Lisa McCurdy, Sheryl Parker, Michael Makani, Julie	2020	A qualitative study on aspects of consent for” genomic research in communities with low literacy	Tanzania	Influence of literacy levels understanding of consent process in genomic studies	BMC Medical Ethics
Bull, Susan Cheah, Phaik Yeong Lwin, Khin Maung Marsh, Vicki Molyneux, Sassy Parker, Michael Theobald, Sally, et al.	2013	Consent and Community Engagement in diverse research contexts: Reviewing and developing research and practice	Multi-country	Interplay between community engagement and researchers- IC process- comprehension, beliefs	Journal of Empirical Research on Human Research Ethics
Burgess, Lesley Jean Gerber, Berna Coetzee, Kathleen Terblanche, Marli Agar, Gareth Kotze, Theunis Jvw	2019	An evaluation of informed consent comprehension by adult trial participants in South Africa at the time of providing consent for” clinical trial participation and a review of the literature	South Africa	Assessment of participants’ comprehension of IC process in a clinical trial; strategies to improve comprehension	Open Access Journal of Clinical Trials
Campbell, Megan M. Susser, Ezra Mall, Sumaya Mqulwana, Sibonile G. Mndini, Michael M. Ntola, Odwa A. Nagdee, Mohamed Zingela, Zukiswa Van Wyk, Stephanus Stein, Dan J.	2020	Using iterative learning to improve understanding during the informed consent process in a South African psychiatric genomics study	South Africa	Assess research predictor’s for” better understanding of the IC- development of standardised tool to improve comprehension	PLoS ONE
Chaisson, Lelia H. Kass, Nancy E. Chengeta, Bafanana Mathebula, Unami Samandari, Taraz	2011	Repeated assessments of informed consent comprehension among HIV-infected participants of a three-year clinical trial in Botswana	Botswana	Assessment of IC comprehension -translation-understand key study information	PLoS ONE
Chapman, K.N Pevzner, E. Mangan, J.M. Breese, P. et al.	2017	Evaluation of the Informed Consent Process of a Multicenter Tuberculosis Treatment Trial	Multi-country	Assessment of tool designed to enhance IC comprehension	AJOB Empir Bioeth.
Chima, Sylvester C.	2013	Evaluating the quality of informed consent and contemporary clinical practices by medical doctors in South Africa: An empirical study	South Africa	Evaluating whether the quality of informed consent obtained by doctors practicing in South Africa is consistent with international ethical standards and local regulations	BMC Medical Ethics
Colom, Marcela Rohloff, Peter	2018	Cultural considerations for” informed consent in paediatric research in low/middle-income countries: A scoping review	Multi-country	Ethical and legal guidelines for” IC in paediatric research; assess cultural and linguistically appropriate strategies for” obtaining IC	BMJ Paediatrics Open
Ditai, J. Kanyago, J. Nambozo, M. R. Odeke, N. M. Abeso, J. Dusabe-Richards, J. Olupot-Olupot, P. Carrol, E. D., er al.	2018	Optimising informed consent for” participants in a randomised controlled trial in rural Uganda: A comparative prospective cohort mixed-methods study	Uganda	Assesses comprehension of study -IC information comparing 3 strategies: standard procedure, slide show and video	BMC
Fischer, A. E. Venter, W. D.F. Collins, S. Carman, M. Lalla-Edward, S. T.	2021	The readability of informed consent forms for” research studies conducted in South Africa	South Africa	Assess readability and comprehension of IC -literacy levels and comprehension of IC	South African Medical Journal
Gikonyo, Caroline Bejon, Philip Marsh, Vicki Molyneux, Sassy	2008	Taking social relationships seriously: Lessons learned from the informed consent practices of a vaccine trial on the Kenyan Coast	Kenya		
Hanrahan, Donna Sexton, Patrina Hui, Katrina Teitcher, Jennifer Sugarman, Jeremy London, Alex John Barnes, Mark, er al.	2015	Linguistic and cultural challenges in communication and translation in US sponsored HIV Prevention research in emerging economies	Multi-country	Assess ethical and regulatory challenges across diverse linguistic and cultural settings	PLoS ONE
Kamaara, Eunice Kong, Camillia Campbell, Megan	2020	Prioritising African perspectives in psychiatric genomics research: Issues of translation and informed consent	Multi-country	Translation of IC in genomic research	Developing World Bioethics
Kiguba, Ronald Kutyabami, Paul Kiwuwa, Stephen Katabira, Elly Sewankambo, Nelson K.	2012	Assessing the quality of informed consent in a resource-limited setting: A cross-sectional study	Uganda	Assessment of quality of IC in clinical and observational studies	
Kithinji, Caroline Kass, Nancy E.	2011	Assessing the readability of non-English-language consent forms: The case of Kiswahili for” research conducted in Kenya	Kenya	Assessed readability and comprehension of IC	IRB:
Koonrungsesomboon, Nut Laothav, Junjira Karbwang, Juntra	2015	Understanding of essential elements required in informed consent form among researchers and institutional review board members	Multi-country	Assessment of the understanding of researchers and members of Institutional Review Boards (IRBs) regarding the essential elements of an Informed Consent Form (ICF)	Tropical Medicine and Health
Krogstad, Donald J. Diop, Samba Diallo, Amadou Mzayek, Fawaz Keating, Joseph	2010	Informed consent in international research: The rationale for” different approaches	Multi-country	Describing strategies to enhance IC comprehension	American Journal of Tropical Medicine and Hygiene
Krosin, Michael T. Klitzman, Robert Levin, Bruce Cheng, Jianfeng Ranney, Megan L.	2006	Problems in comprehension of informed consent in rural and peri-urban Mali, West Africa	Mali	Assessments of challenges in comprehension during consent processes	Clinical Trials
Mack, Natasha Ramirez, Catalina B. Friedland, Barbara Nnko, Soori	2013	Lost in Translation: Assessing Effectiveness of Focus Group Questioning Techniques to Develop Improved Translation of Terminology Used in HIV Prevention Clinical Trials	Tanzania	Assessment of effectiveness of techniques in enhancing participants’ understanding (translations, development of lexicons)	PLoS ONE
Marshall, Patricia A. Adebamowo, Clement A. Adeyemo, Adebowale A. Ogundiran, Temidayo O. Strenski, Teri	2014	Voluntary participation and comprehension of informed consent in a genetic epidemiological study of breast cancer in Nigeria	Nigeria	Assessment of comprehension of IC in genetic study	BMC Medical Ethics
MacQueen, Kathleen M. Chen, Mario Ramirez, Catalina Nnko, Sooriea Earp, Kelly M.	2014	Comparison of closed-ended, open-ended, and perceived informed consent comprehension measures for” a mock HIV prevention trial among women in Tanzania	Tanzania	Assessment of strategies to enhance comprehension of IC	PLoS ONE
Mamotte, Nicole Wassenaar, Douglas Koen, Jennifer Essack, Zaynab	2010	Convergent ethical issues in HIV/AIDS, tuberculosis and malaria vaccine trials in Africa: Report from the WHO/UNAIDS African AIDS Vaccine Programme’s Ethics, Law and Human Rights Collaborating Centre consultation, 10–11 February 2009, Durban, South Africa	Multi-country	Description of factors influencing the comprehension of IC	BMC Medical Ethics
Mandava, Amulya Pace, Christine Campbell, Benjamin Emanuel, Ezekiel	2016	The quality of informed consent: mapping the landscape. A review of empirical data from developing and developed countries	Multi-country	Evaluation of IC comprehension in developed and developing countries s	J Med Ethics.
Minnies, Deon Hawkridge, Tony Hanekom, Willem Ehrlich, Rodney London, Leslie	2008	Evaluation of the quality of informed consent in a vaccine field trial in a developing country setting	South Africa	Assessment of the quality of consent in the case control study, and to identify factors that may influence the quality of consent.	BMC Medical Ethics
Moodley, K. Pather, M. Myer, L.	2005	Informed consent and participant perceptions of influenza vaccine trials in South Africa	South Africa	Assessment of knowledge and perceptions of the informed consent process among individuals participating in influenza vaccine trials in two disadvantaged communities in	Journal of Medical Ethics
Munung, Nchangwi Syntia Marshall, Patricia Campbell, Megan Littler, Katherine Masiye, Francis	2016	Obtaining informed consent for genomics research in Africa: Analysis of H3Africa consent documents	Multi-country	Assessment of consent models used in genomic research	Journal of Medical Ethics
Muzanyi, Grace Sekitoleko, Isaac Johnson, John L. Lunkuse, Jane Nalugwa, Gladys., er al.	2020	Level of education and preferred language of informed consent for” clinical research in a multi-lingual community	Uganda	Assessment of educational level and preferred language of consent	African Health Sciences
Nnabugwu, Ikenna I. Ugwumba, Fredrick O. Udeh, Emeka I. Anyimba, Solomon K. Ozoemena, Oyiogu F.	2017	Informed consent for” clinical treatment in low-income setting: Evaluating the relationship between satisfying consent and extent of recall of consent information	Nigeria	Assessment of factors that influence comprehension and information recall during IC	BMC Medical Ethics
Odhiambo, Rachael Mars, Maurice	2018	Patients’ understanding of telemedicine terms required for” informed consent when translated into Kiswahili	Kenya	Assessment of patients’ understanding of translated IC documents	BMC Public Health
Oduro, Abraham R. Ab”OR”igo, Raymond A. Amugsi, Dickson Anto, Francis Any”OR”igiya, Thomas., er al.	2008	Understanding and retention of the informed consent process among parents in rural northern Ghana	Ghana	Assessment of the comprehension and retainment of information of the informed consent process	BMC Medical Ethics
Olanrewaju, Muhammed	2014	Multimedia Informed Consent Tool for” a Low Literacy African Research Population: Development and Pilot-Testing	Gambia	Evaluation of a multimedia informed consent tool for” people with low literacy in an area where a malaria treatment trial	Journal of Clinical Research & Bioethics
Ossemane, Ezaquiel, B. Moon, Try, D. Sacarlal, J. Sevene, Esperanca Kenga, Darlene., er al.	2016	Assessment of Parents’/Guardians’ Initial Comprehension and One-Day Recall of Elements of Informed Consent within a Mozambican Study of Pediatric Bacteremia	Mozambique	Evaluation of tool used to enhance the comprehension of the elements of informed consent by the parents/guardians of children enrolled in a clinical study	J Empir Res Hum Res Ethics.
Palmeirim, Marta S. Mohammed, Ulfat A. Ross, Amanda Ame, Shaali M. Ali, Said M.	2021	Evaluation of two communication tools, slideshow and theatre, to improve participants’ understanding of a clinical trial in the informed consent procedure on Pemba Island, Tanzania	Tanzania	Assessment of communication strategies to enhance IC comprehension	PLoS Neglected Tropical Diseases
Palmeirim, Marta S. Ross, Amanda Obrist, Brigit Mohammed, Ulfat A. Ame, Shaali M.	2020	Informed consent procedure in a double blind randomized anthelminthic trial on Pemba Island, Tanzania: Do pamphlet and information session increase caregivers knowledge?	Tanzania	Assessment of strategies- pamphlets, oral information and combination of pamphlets and oral information in improving participants knowledge about study information	BMC Medical Ethics
Penn, Claire Evans, Melanie	2010	Assessing the impact of a modified informed consent process in a South African HIV/AIDS research trial	South Africa	Assessment of comprehension of IC using standard and modified IC documents	Patient Education and Counseling
Reynolds, Lindsey Cousins, Thomas Newell, Marie Louise Imrie, John	2013	The social dynamics of consent and refusal in HIV surveillance in rural South Africa	South Africa	Assessment of consent encounter in HIV surveillance study	Social Science and Medicine
Ssali, Agnes Poland, Fiona Seeley, Janet	2015	Volunteer experiences and perceptions of the informed consent process: Lessons from two HIV clinical trials in Uganda Ethics in Biomedical Research	Uganda	Assessment of participants understanding of IC; strategies for” improving IC process	BMC Medical Ethics
Ssali, Agnes Poland, Fiona Seeley, Janet	2016	Exploring informed consent in HIV clinical trials : A case study in Uganda	Uganda	Assessment of key actor’s during IC process	HLY
Staunton, Ciara	2015	Informed consent for” HIV cure research in South Africa: Issues to consider	South Africa	Issues to consider to improve comprehension of IC	BMC Medical Ethics
Staunton, Ciara de Roubaix, Malcolm Baatjies, Dianno Black, Gill Hendricks, Melany. et al.	2018	Ethical challenges in developing an educational video to empower potential participants during consent processes in HIV cure research in South Africa	South Africa	Challenges in use of video to enhance IC process	BMC Medical Ethics

### Social and cultural contexts

We found a recognition on the interplay between socio-cultural context and the IC process [9–15]. Nevertheless, Marshall [13] and Reynolds et al. [5] show that aligning legal-ethical principles and socio-cultural realities remain a challenge. Key issues include challenges to the way the ethical guidance approaches IC. Krogstad et al. [16], suggest that international guidelines place too much emphasis on the importance of the individual in the consent process. Therefore, regulatory IC requirements could violate the core individual ethics principles of participants. Following this type of reasoning in many SSA settings it is submitted that the IC process needs to be multi-layered involving family and community members [17–21]. The review by Krogstad [16], and a study in Tanzania by Palmeirim [22], showed that proper consideration of a social context approach to consent meant communities in rural settings place high value on oral interactions. Verbal consent is commonly obtained often in the presence of a literate witness who is able to read available consent documents. However, Colom [10] notes that this raised concerns that the witnesses may impose their views on the consenting participant or be selective about the information offered rather than encouraging dialogue and acting as a safeguard. Ssali et al. [23] in Uganda, argued that obtaining a volunteer’s signature or thumbprint on a consent form raised issues of trust and did not necessarily enhance IC comprehension [24]. Nevertheless, this approach brings with it its own complexities. Fourthly, studies in Kenya (Boga [23], Ghana (Tindana [25] and South Africa (Zulu [26] report difficulties with the definition of community, competing interests, social and power inequities and the impact these have on participants’ comprehension of the IC process. Fifthly, there was little guidance on how this multi-layered approach could be implemented.

### Gaps and inconsistencies in the ethical and legal frameworks guiding the informed consent process

Although there is a recognition that IC must be understood within the specific social and cultural context, the normative ethical-legal framework was found to be lacking. Two main issues emerged from this review. First, there are contradictions between national and international norms in respect to the requirements for comprehension during the IC process [10, 27]. Inconsistent legal norms were compounded by variations between the legal and ethical guidelines within countries. Four studies by Colom [10], Andrews [28] Matimba et al. [29] and Wright et al. [30] highlight these complexities, including a lack of consistency about what information is essential for research participants to know and a lack of regulatory guidance and language for the collection and use of human biospecimens in many SSA countries Barchi [31]. Secondly, the lack of detail in how to operationalise the core aspects of IC. These concerns add a layer of complexity for researchers [10, 27].They have also led to a growing interest in updating and aligning country-specific guidelines with the law to ensure that research participants are adequately protected.

### Strategies used to improve participants’ understanding of IC

In the papers reviewed, we identified four categories of interventions used to improve comprehension in the consent process. These included translation of consent forms [7, 23–32], and multimedia medium [21, 33–39], IC assessment tools and a combination of these strategies [40].

### The language used and the translation of IC

Many SSA countries are characterised by multilingual communities. (Supplementary Table 2: Languages spoken in SSA countries reviewed). This led to two inter-related issues with the language in IC forms; the words or terms used by local communities and the translation of them into local languages to make them more accessible. The studies reviewed reported misunderstandings and miscommunication, especially when investigators and participants speak different languages, when IC documents have to be translated, or when scientific research and the notion of IC are unfamiliar to study participants [33, 37, 40, 42, 49–53].

The nature of the words used in IC are critical to understanding. Terms such as ‘understanding’, ‘comprehension’, ‘knowledge’, ‘remembering’, ‘retention’, ‘recall, ‘awareness’ or ‘recognition’ were used interchangeably with the potential to influence apprehension of the information. Interestingly, Bentley et al. [41] attempted to make IC more accessible by the incorporation of culturally appropriate analogies, a method linked to a theory for improving IC comprehension in Malawi.

Although it is accepted that the translation of IC forms into local languages is essential it brings with it a range of changes. Studies by Palmeirim [22, 42], Afolabi et al. [35, 43, 44], Mack et al. [45], Staunton [46], Penn [47], and Moodley et al. [48]reported the challenges of providing information in the participants’ native languages because in many communities, local languages exist only in oral forms and they do not have standardised writing formats. This made written translation and back-translations of informed consent documents not only impractical, but also less precise, and may inadvertently misrepresent the research being conducted. Despite this, studies across SSA countries primarily employ back-translation. To address limitations inherent in translations, Boga and colleagues in Kenya [23], went beyond translations and targeted the broader socio- cultural context by co-developing a dictionary of language with communities to incorporate the socio-cultural nuances and issues that might be missed during the translation. Interestingly, only one study by Baiden and colleagues [49]argued against the appropriateness of translating approved English version ICF into the local language, and instead proposed the development of contextualized informed procedures based on the values and aspirations of the participants in different contexts. In fact, Burgess et al. argued that translating IC documents into unfamiliar local dialects could ironically enhance the vulnerability of the participants [50].

A study by Muzanyi et al. [51] found that the participants’ choice of language was associated with the level of education in Uganda, a preference for English may be influenced by English being one of the national mediums of communication. In Tanzania, Bukini and colleagues [38] reported that low literacy levels had little influence on comprehension of IC, rather, the methods used to provide information, the language, and time spent with the study participants were the key factors influencing understanding.

### IC assessment tools

We identified a range of IC assessment tools, strategies and approaches employed to improve comprehension of IC forms. The tools ranged from study quizzes, psycho-metric development and testing tools through to multimedia interventions [33, 37, 52–56]. The assessment methods used differed significantly, ranging from recall and retention of specific elements of the IC, readability of IC forms, overall assessment of language and meaning, as well as participants’ satisfaction about the IC process [57, 50]Morrow et al. [52] focusing on research into paediatric critical care revealed concerns about therapeutic misconceptions in medical research [58]. In this study, Morrow and colleagues showed that most participants in South Africa perceive medical research to be similar to medical care, and may not understand the study purpose and therefore caregivers believed that their infants would be protected from HIV if they joined the research project. Similar findings were reported by Moodley et al. [48]In Ghana, Baiden et al. [49], Malawi, Bentley [59]South Africa, Ndebele et al. [41], and Moodley et al. [60] reported that most clinical trials with complex study procedures or consent forms tended to evaluate the understanding or recall of specific scientific and technical trial terms including randomisation, placebo. In Ghana, O [57] reported varied comprehension levels of disclosed information among participants and variability was also observed among younger and older participants. In Mozambique and South Africa, Ossemane et al. [61] and Fischer et al. [62], indicated that readability of IC was influenced by long sentences, the number of words containing three or more syllables of words per sentence resulting in poor comprehension. In South Africa the study by Fischer et al. [62] showed that two-thirds of the ICFs analysed for readability did not meet recommendations by the national ethical guidelines stipulations, and that the IC documents were hard to read and exceeded the South African national functional literacy level of grade 7, equivalent to end of primary school level education. Afolabi et al. [39] used digitised audio tools in the participants’ local languages to enhance comprehension among clinical trial participants with low-literacy levels in Gambia. While most studies focused on the adult population, in a study in Kenya, Afolabi and colleagues [33] adapted the assessment tool (DICCQ) among a diverse population of adolescents, young adults and parents.

Findings on assessment tools showed varying degrees of efficacy, and there were suggestions that these tools are inadequate [40, 41, 48]. Ssali et al., [23] working in Uganda pointed out that while the assessment tools are important, the language used may be more important in enhancing IC comprehension [24]. Afolabi et al. [43] note that empirical assessment of consent comprehension in many SSA countries is in its infancy and that the means of assessing understanding may be unfamiliar and confusing for participants. Afolabi and colleagues further highlighted that the paucity of studies on instruments for informed consent comprehension is not surprising, given the cost and highly technical nature of psycho-metric development and testing of a comprehension instrument.

## Discussion

Our review showed wide recognition in published studies that the process of achieving IC in SSA is fraught with challenges. We also showed complexities of the languages in multilingual settings and the limitations of translating IC documents to make them accessible to local languages. Furthermore, we underscore the importance of addressing social and cultural contexts in the informed consent process, as well as the complexities of operationalising IC documents in a culturally appropriate manner. For example, the insistence on written communications in settings where communities value oral communication and signing of the IC does not enhance or guarantee comprehension. Rather, these practices are conducted to meet legal requirements. Consequently, most of the studies suggest a need for flexibility and negotiations around the norms to suit the diverse socio-cultural contexts.

The current normative guidelines have gaps and inconsistencies [27, 30]. One of the points of conflict between ethics and law is around obtaining informed consent in social and cultural contexts where decision-making is not solely in the hands of an individual. Following a legal approach ethical guidelines tend to also follow an individualistic approach to consent leaving limited guidance on how to work in social contexts where this notion is foreign. The presence of ambiguity in the legal and ethical frameworks that govern the IC process is not limited to SSA, but exists globally [16]. One gap is the need to develop guidelines that define the most crucial information relevant for comprehension of informed consent in SSA research settings as well as the best way of how this information should be communicated. A further concern is the focus on individual consent is a contested position with varying views on how one can meet the need for individual autonomy with the cultural context in certain communities. Given this lack of consensus on the guidelines, operationalising them is difficult.

Similar findings have been reported across the globe, for example in the Asia-Pacific region [4, 63–65], United States [66–71], and Europe [72, 73]. However, the complexities tend to be more pronounced in SSA countries due to among others; the socio-cultural context, poverty and power relations. Studies reviewed showed that although high rates of illiteracy and functional illiteracy may contribute to the difficulties of comprehension of IC, the language and delivery of the IC information ranked high in the barriers to IC [3, 39, 74–76]. Concerningly, most of the studies reported on the participants’ performance and few focused on the researchers’ communication skills and delivery of IC process, a key factor during the IC process. Our review showed that similar to the study participants, most researchers are equally ill prepared and often have a limited understanding of the legal and clinical terms during the IC process [7, 32, 74–78]. This may represent an asymmetry, with the emphasis on the failure of the participants without focusing on the role of the researcher’ communication and delivery skills. We also showed limitations and challenges inherent in the language used and the translation of IC, and assessment tools aimed at enhancing comprehension of the IC process.

Our review showed that comprehension of IC in paediatric research is under-represented in SSA despite wide support for adolescent participation in health research.

Although we set out to identify strategies to enhance compression of the IC process, our review showed that most the strategies used including translation and backtranslations; tools developed to assess and enhance comprehension of the IC process do not address the structural, systematic and contextual issues that impact the IC process and directly affect understanding. Considering these limitations and complexities inherent in the IC consent process, we suggest alternative approaches moving beyond translations of the literal language and efforts to seek to address contextual and structural factors that impact comprehension of the IC process. This would include locating the participants’ world view at the centre of the IC process and taking account of how they perceive the IC process instead of the top-down approach in which the participants fit in with the process devised by others. This requires researchers, and ethics committee members to reflect and ask questions such as does the signing of the IC documents equate to comprehension, how can linguistic and contextual factors be integrated to ensure valid consent, and what researcher’ factors impact delivery of IC process and what strategies can be implemented to mitigate highlighted gaps in varied contexts.

### Strengths ad limitations

One of the strengths of the review is that it advances the discussion regarding IC comprehension beyond the limitations of the assessment and translation of IC documents and suggests perspectives that researchers should consider enhancing the IC process. These include: moving beyond the literal language and translations to understanding IC comprehension from the participants’ perspectives, as well as examining researcher factors that impact the IC process.

One of the limitations is that we did not assess the domains of the various elements such as voluntary participation, compensation, confidentiality, anonymity, risks and benefits. Furthermore, studies which assessed the domains of IC documents varied considerably with little regard to the crucial information that could engender comprehension.

## Conclusion

We conducted a scoping review to examine published studies focusing on improving the IC process and assessing impact of these strategies in sub-Saharan Africa countries. Our review showed that while translations of IC documents and assessment tools improve comprehension of IC documents, these strategies continue to face limitations and challenges, and do little to address the underlying socio-cultural factors that constrain comprehension of the IC process. Our review suggests that there is a need for greater flexibility and negotiations with communities to ensure that the approach to IC is suited to the diverse socio-cultural contexts.

## Supplementary Information

**Additional file 1: Table 1.** Thematic analysis of studies reviewed.

**Additional file 2: Table 2.** Languages spoken in SSA countries reviewed.

## Data Availability

Not applicable.

## Abbreviations

IC: Informed consent
LMIC: Low and middle income countries
SSA: Sub-Saharan Africa
