# Supplementary material for: Beyond translations, perspectives for researchers to consider to enhance comprehension during consent processes for health research in sub-saharan Africa: a scoping review
Source: BMC Med Ethics. 2023 Jun 21;24:43. doi: 10.1186/s12910-023-00920-1 (PMC10286482; doi:10.1186/s12910-023-00920-1)
Supplement: Supplementary file 1 — Additional file 1: Table 1. Thematic analysis of studies reviewed. [file 12910_2023_920_MOESM1_ESM.docx]

**Supplementary file Table 1: Thematic analysis of studies reviewed**

| Theme | Sub-theme | Challenges in IC | Country |
| --- | --- | --- | --- |
|  |  |  |  |
| Social and cultural contexts | Individual versus relational decision making practices | - Poor alignment between socio-cultural context and ethical-legal framework^[20, 5]^ - Emphasis on individual versus relational approach to IC process^[23-28,32]^ - Rural communities value communication compared to written communication[^33]^ - Individuals likely to participate to appease community leaders or gatekeepers without adequate comprehension of the IC process^[29-31]^ | Multi-country, South Africa  Multi-country study, Kenya, Ghana, Uganda, Tanzania  Tanzania  Ghana, Kenya, Swaziland, South Africa |
|  | Power structures | Presence of a witness may influence participants views than enhance comprehension^[25,34]^ | Uganda |
| Gaps and inconsistencies in the ethical and legal frameworks guiding the informed consent process | International and national frameworks | - Lack of clarity in international and national ethical guidelines that govern IC^[10-12]^ - Inconsistencies in various guidelines within a country[^10-16]^ | Ghana, Kenya, Mozambique, South Africa  Multi-country sites, Kenya, South Africa, Mozambique, Zambia |

| Strategies used to improve participants’ understanding of IC | Language | - Multiple languages with diverse dialects -different languages spoken by the researchers and participants is likely to compound confusion and misunderstanding; using terms and words with different meaning interchangeably^[35, 36, 42, 44, 58–61]^ | Multi-country sites, Botswana, Gambia, Kenya, Malawi, Mozambique, South Africa, Uganda |
| --- | --- | --- | --- |
|  | Translation of IC documents | - Complexities in translating scientific and technical terms into local dialects without standardised written form compound confusion of IC documents^[35,36,42,44,58-61]^ | Multi-country sites, Botswana, Kenya, Malawi, Mozambique, South Africa, Uganda |
|  |  | - Low functional literacy levels and poor understanding of scientific and technical terms ^[48]^ | Tanzania |
|  | IC assessment tools | - Assessment tools focus on knowledge retention, recall instead of IC comprehension^[36,47,50-52]^ - Assessment methods unfamiliar and confusing to the participants^[36]^ - Variability in methods used, and purpose of assessment and degrees of efficacy in improving IC comprehension^(49)^ - High costs associated with assessment tools^[36]^ | Kenya, Uganda, South Africa  Kenya  Gambia  Multi-country sites, Kenya |
|  | Functional literacy levels | - IC documents were hard to read and exceeded participants’ functional literacy levels^[11,57]^ | Mozambique, South Africa |
