# Supplementary material for: Beyond translations, perspectives for researchers to consider to enhance comprehension during consent processes for health research in sub-saharan Africa: a scoping review
Source: BMC Med Ethics. 2023 Jun 21;24:43. doi: 10.1186/s12910-023-00920-1 (PMC10286482; doi:10.1186/s12910-023-00920-1)
Supplement: Supplementary file 2 — Additional file 2: Table 2. Languages spoken in SSA countries reviewed. [file 12910_2023_920_MOESM2_ESM.docx]

Supplementary file Table 2: Languages spoken in SSA countries reviewed

| Country | Official languages | Spoken/native languages |
| --- | --- | --- |
| Botswana | English and Setswana | 31 (26 indigenous and 5 non-indigenous languages) |
| Gambia | English | 10 |
| Ghana | English | 50 |
| Kenya | English and Swahili | 68 |
| Malawi | English | 16 |
| Mali | French | 15 |
| Mozambique | Portuguese | 40 |
| Nigeria | English | 20 major languages |
| South Africa | isiZulu, Sepedi, Setswana, Siswati, Tshivenda, Xistonga, Afrikaans, English, siNdebele, iSixhosa | 11 |
| Tanzania | kiSwahili, Arabic, English | 125 |
| Uganda | Luganda, Swahili, English | 41 |
